# Lack of Benefit of Early Intervention with Dietary Flax and Fish Oil and Soy Protein in Orthologous Rodent Models of Human Hereditary Polycystic Kidney Disease

**DOI:** 10.1371/journal.pone.0155790

**Published:** 2016-05-23

**Authors:** Tamio Yamaguchi, Jessay G. Devassy, Md Monirujjaman, Melissa Gabbs, Harold M. Aukema

**Affiliations:** 1 Department of Human Nutritional Sciences, University of Manitoba, Winnipeg, MB, Canada; 2 Canadian Centre for Agri-Food Research in Health and Medicine, St Boniface Hospital Research Centre, Winnipeg, MB, Canada; 3 Department of Clinical Nutrition, Suzuka University of Medical Science, Suzuka, Mie, Japan; 4 Children’s Hospital Research Institute of Manitoba, Winnipeg, MB, Canada; UCL Institute of Child Health, UNITED KINGDOM

## Abstract

Rationale for dietary advice in polycystic kidney disease (PKD) is based in part on animal studies that have examined non-orthologous models with progressive development of cystic disease. Since no model completely mimics human PKD, the purpose of the current studies was to examine the effects of dietary soy protein (compared to casein) or oils enriched in omega-3 fatty acids (fish or flax oil compared to soy oil) on early disease progression in two orthologous models of PKD. The models studied were *Pkd2*^WS25/-^ mice as a model of autosomal dominant PKD, and PCK rats as a model of autosomal recessive PKD. After 13 weeks of feeding, dietary fish (but not flax) oil resulted in larger kidneys and greater kidney water content in female *Pkd2*^WS25/-^ compared to control mice. After 12 weeks of feeding male PCK compared to control rats, both fish and flax compared to soy oil resulted in enlarged kidneys and livers, greater kidney water content and higher kidney cyst area in diseased rats. Dietary soy protein compared to casein had no effects in *Pkd2*^WS25/-^ compared to control mice. In PCK rats, kidney and liver histology were not improved, but lower proteinuria and higher urine pH suggest that soy protein could be beneficial in the long term. Therefore, in contrast to studies in non-orthologous models during the progressive development phase, these studies in orthologous PKD models do not support dietary advice to increase soy protein or oils enriched in omega-3 oils in early PKD.

## Introduction

Hereditary polycystic kidney disease (PKD) is characterized by countless renal cysts and often also displays significant liver cysts. The two major types of PKD are autosomal dominant PKD (ADPKD) and autosomal recessive PKD (ARPKD). Approximately 80–85% of ADPKD is caused by mutations in *PKD1*, the gene for polycystin 1, and the remaining 15–20% of cases are caused by mutations in *PKD2*, which codes for polycystin 2 [1]. ARPKD is much less common but is a more severe form of disease that primarily affects the pediatric population. ARPKD is caused by a mutation in the polycystic kidney and hepatic disease-1 (*PKHD1*) gene, which codes for polyductin/fibrocystin [2].

Despite the promise of pharmacological treatments such as vasopressin receptor antagonists, the presence of undesirable side effects [3] and the lack of efficacy of current treatments in delaying the need for renal replacement therapy [4], has led to much interest in alternative treatments such as dietary therapy. This is evidenced by ADPKD diet studies currently in progress [5,6], as well as nutritional advice on several national PKD association web pages addressing nutrition questions [7–9]. In their dietary advice, PKD Foundations in both Canada and the US cite animal studies as evidence of the potential effectiveness of dietary soy and plant proteins. Dietary soy protein has been particularly effective in several spontaneous rodent models of renal cystic diseases such as the Han:SPRD-*Cy* rat with the mutated *Anks6* (formally called *Pkdr1*) gene and the *pcy*/*pcy* (*pcy*) mouse that harbors the *pcy* mutation [10–16]. In these models, renal cyst disease develops progressively and soy protein feeding resulted in lower kidney size and water content, along with reduced cyst growth and fibrosis, when replacing casein as the protein source in the standard AIN93 laboratory rodent diet [17].

The PKD Foundations in Canada and the US also advise patients to consume omega-3 fatty acids, again based on animal model data. Indeed, dietary flax oil (enriched in alpha-linolenic acid) reduces kidney size, water content, cyst growth and fibrosis in both the Han:SPRD-*Cy* rat and the *pcy* mouse [18–22]. However, fish oil (enriched in EPA and DHA) appears to have beneficial effects in the Han:SPRD-*cy* rat [23,24], but not always in the *pcy* mouse [21,25–27].

In both dietary soy protein and omega-3 oil interventions, male animals have been used, as they typically display greater disease progression. However, recent studies in the female PCK rat model of ARPKD have suggested that dietary soy protein or fish oil may not be effective in this orthologous model of ARPKD [28]. Further, the effects of dietary soy protein or omega-3 oils in orthologous models of ADPKD have not yet been determined in either males or females, nor have male PCK rats been tested with these dietary treatments. Therefore, the effects of soy protein compared to casein and flax or fish oil compared to soy oil were examined in the early stages of disease in males and females of an orthologous mouse model of ADPKD (*Pkd2*^ws25/-^ mice) and in the male PCK rat model of ARPKD.

## Materials and Methods

All animal procedures were approved by the University of Manitoba Animal Care Committee and adhered to the guidelines of the Canadian Council on Animal Care.

### *Pkd2*^ws25/-^ mice

*Pkd2*^ws25/ws25^ and *Pkd2*^+/-^ breeders were obtained from Dr. Stefen Somlo at Yale University (New Haven, CT, U.S.A.) [29]. These genotypes were crossed to produce mice with diseased (*Pkd2*^ws25/-^) or normal (*Pkd2*^ws25/+^) phenotypes. All mice were given diets based on the American Institute of Nutrition (AIN) 93G standard diet for laboratory rodents [17], which has casein as the standard protein source and soy oil as the standard oil. The experimental diets contained either an equivalent amount of soy protein that replaced the casein, or either flax oil or fish oil that replaced 80% of the soy oil, as shown in S1 Table and detailed in previous studies of non-orthologous models of PKD [11,21]. All oils and diet ingredients were purchased from Dyets Inc. (Bethlehem, PA, USA). The oils contained 0.02% tBHQ to prevent oxidation and diet ingredients were stored at 4°C. Diet was freshly prepared twice per month and stored in sealed containers at -20°C until feeding. Routine examination of texture, odor, and color indicated that the oils were not oxidized. Mice were housed singly in cages with plastic enrichment domes in a temperature- and humidity-controlled environment with a 12 hour day/night cycle and were given free access to water and diet.

The feeding period was for 13 weeks, from 3 to 16 weeks of age, and feed and water disappearance were determined during week 6 of feeding to estimate feed and water intakes, respectively. Mice were monitored daily and no mice became ill or died. Mice were anesthetized to surgical plane using isofluorane and euthanized via exsanguination. Normal and diseased mice were identified by the absence or presence of renal cysts. Body, kidney and liver weights were recorded before placing the left kidney and a portion of the liver in 10% formalin for 24h, followed by transfer to PBS at 4°C until further processing. The right kidney and another portion of the liver were snap frozen in liquid nitrogen, and lyophilized to determine tissue water content.

Formalin fixed kidneys and livers were embedded in paraffin, sectioned at 5 μm and tissue sections were stained with Masson's trichrome to measure cyst and fibrosis area as previously described [21,24]. A Nikon D600 FX DSLR camera equipped with a 60mm F2.8 Macro lens (Nikon Corporation, Mississauga, Canada) was used to capture images of backlit whole kidney sections. Macro rings were used between the camera body and lens to achieve 2.5X magnification. This allowed clear identification of open spaces from complete coverage of the kidney or liver sections in each picture. Quantitative analysis of cyst area of the whole kidney section and the sample liver section was performed using Image Pro software (Media Cybernetics, Silverspring, MO), after coloring in the white areas of tubular lumen spaces to eliminate these from measurement. In addition to cyst area, the blue areas in stained sections were used to examine fibrosis by densitometry as previously described [21,24].

### PCK rats

Weanling male PCK rats were purchased from a commercial breeder (Charles River, QC, Canada) and normal and diseased rats were provided the same diets and housing conditions as described for study 1. PCK rats were fed these diets for 12 weeks, from 4 to 16 weeks of age, and feed and water disappearance determined during week 10 of the feeding period using metabolic cages. Rats were monitored daily and one rat in the casein fish oil group was euthanized in week 2 due to poor overall condition and was found to have an enlarged heart upon autopsy. A second rat in the casein fish oil group was terminated in week 12 due to excessive weight loss and poor condition, and was found to have enlarged kidneys, liver and spleen upon autopsy. Rats were anesthetized as described for mice. At termination, tissues were processed as described above for *Pkd2*^ws25/-^ tissues, with the exception of cyst area measurements, for which a Nikon D90 DX DSLR camera was used. Tubular lumen spaces could not be accurately differentiated from cysts in these sections, so white spaces from both the cysts and tubular lumen spaces in these sections were quantified together. Serum and urinary creatinine were measured using the Jaffe reaction as modified by Heinegard & Tiderstrom and adapted for micro assay [21]. Creatinine clearance was calculated using urine volume and urine creatinine from week 10 of the feeding period and serum creatinine from termination. Urine pH was measured immediately after 24 hour urine collection and urine protein was determined using the Bradford protein assay method with bovine serum albumin as a standard [30]. Blood pressure was measured in conscious rats at week 12 of feeding period using a multichannel blood pressure system with a tail-cuff sphygmomanometer (Coda 6 System, Kent Scientific, Torrington, Conn), as described [31].

### Statistical Analyses

To first determine effects of disease and sex, only those provided soy oil (control oil) were compared. In *Pkd2*^WS25/-^ and *Pkd2*^WS25/+^ mice, 3-way ANOVA (sex x disease x protein) revealed that there were no protein effects on disease, so mice given soy protein and casein were combined for analyses by 2-way (disease x sex) ANOVA. For PCK rats, only males were used, so disease effects in the soy oil fed rats were tested using t-tests.

Dietary effects were tested in *Pkd2*^WS25/-^ animals only, and 3-way ANOVA (sex x oil x protein) again revealed that protein had no effect on any parameters. Therefore, mice given soy protein and casein were combined and a 2-way ANOVA (oil x sex) was performed. For PCK rats 2-way ANOVA (oil x protein) was used, as only males were used for this study.

All ANOVA were performed using the GLM procedure of SAS (SAS, version 9.3, Cary, NC) followed by Duncan’s Multiple Range test to delineate significant oil or interaction effects. Normality of the data was assessed using the Shapiro–Wilk's test, and non-normal data was normalized by log transformation where possible. If normality was not achieved, data were analyzed using the Kruskal–Wallis test. Statistical significance for main and interaction effects was set at P < 0.05. All data are presented as mean±SE.

## Results

### *Pkd2*^WS25/-^ mice

**Fig 1:**
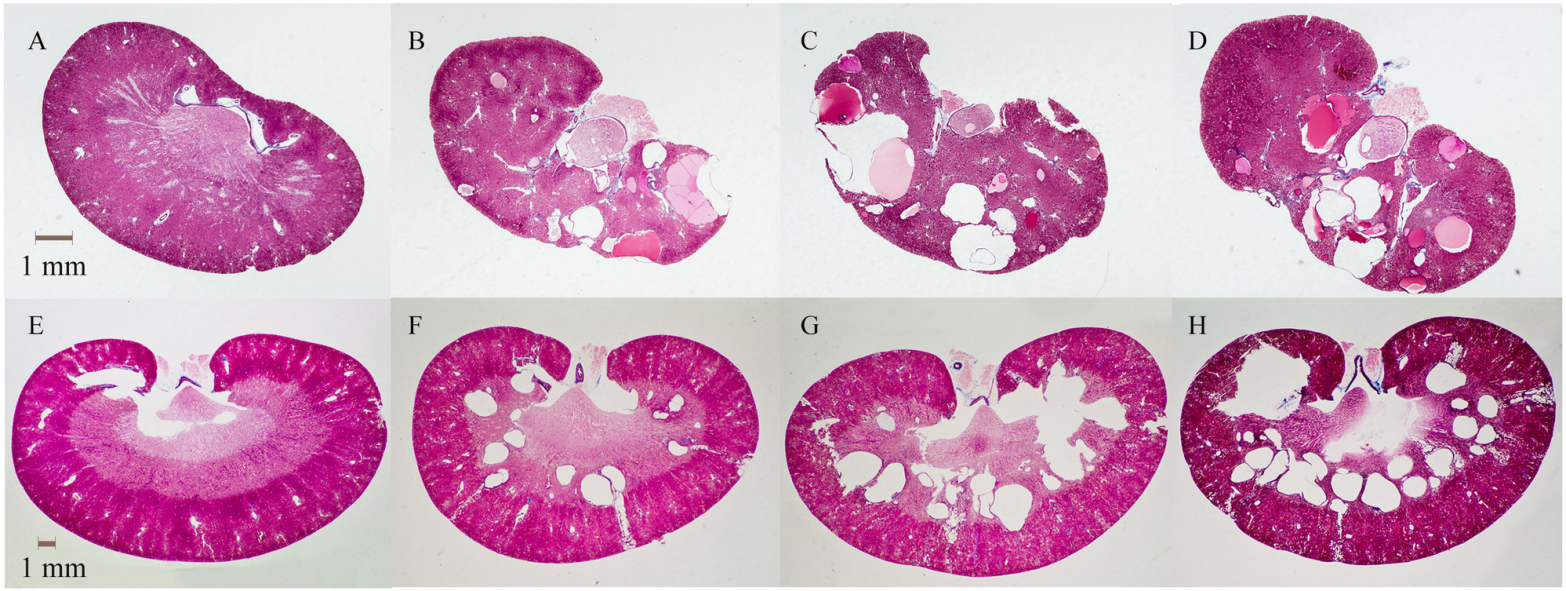
Kidney sections. Sections A-D are from Pkd2 mice and E-F are from PCK rats as follows: (A) *Pkd2*^WS25/+^ (normal) and (B) *Pkd2*^WS25/-^ (diseased) mice provided soy oil, *Pkd2*^WS25/-^ (diseased) mice provided (C) flax oil or (D) fish oil, (E) normal and (F) PCK rats provided soy oil, PCK rats provided (G) flax oil or (H) fish oil. Scale bar = 1 mm.

**Fig 2:**
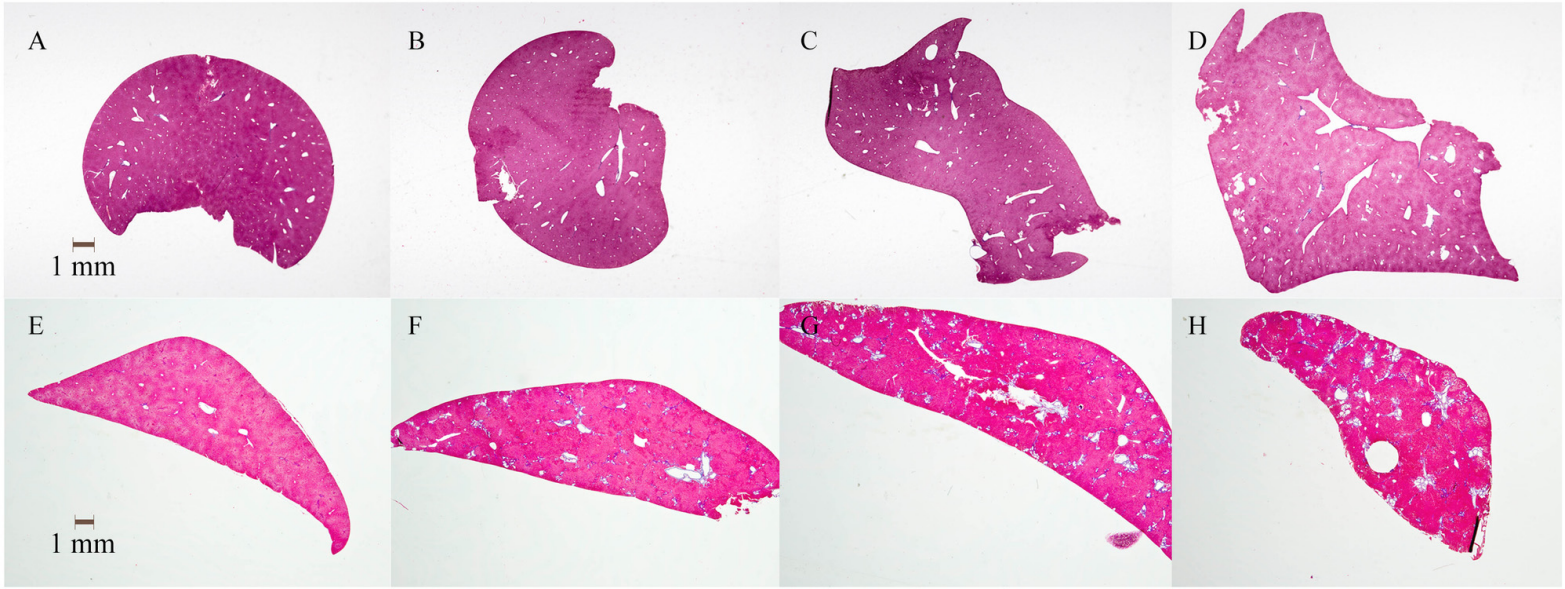
Liver sections. Sections A-D are from Pkd2 mice and E-F are from PCK rats as follows: (A) *Pkd2*^WS25/+^ (normal) and (B) *Pkd2*^WS25/-^ (diseased) mice provided soy oil, *Pkd2*^WS25/-^ (diseased) mice provided (C) flax oil or (D) fish oil, (E) normal and (F) PCK rats provided soy oil, PCK rats provided (G) flax oil or (H) fish oil. Scale bar = 1 mm.

At the end of the feeding period, the area comprising both cysts and tubular lumen made up ~15–20% of the kidney section areas in *Pkd2*^WS25/-^ mice (Fig 1A–1D). On the other hand, livers displayed fewer and smaller cysts, with 42% of mice exhibiting no cysts at all, and none displaying significant fibrosis (Fig 2A–2D). As well, 3-way (sex x disease x protein) ANOVA of the soy oil fed mice revealed that there was no protein effect on any of the parameters measured, indicating no benefit of soy protein compared to the casein protein. The protein groups were therefore combined, and in mice provided the soy oil diets, cyst development resulted in higher kidney weights and water content in diseased compared to normal mice (Table 1). Consistent with the small and sporadic liver cysts observed, liver weights were not elevated in diseased compared to normal mice. Body and tissue weights were higher in males compared to females, but there were no sex differences in any other parameters (Table 1).

**Table 1:** Disease and sex effects in *Pkd2*^WS25/-^ and *Pkd2*^WS25/+^ mice.

	*Pkd2*^WS25/+^		*Pkd2*^WS25/-^			
	Male	Female	Male	Female	P<0.05	
Body weight (g)	26.2±2.2	19.5±0.6	27.1±1.7	19.3±1.0		S
Kidney						
weight (g)	0.28±0.02	0.21±0.01	0.37±0.04	0.27±0.03	D	S
weight / body weight (g/100g)	1.05±0.04	1.11±0.04	1.37±0.01	1.40±0.12	D	
water content (%)	72.2±0.6	71.5±1.0	75.4±1.8	74.8±1.1	D	
cyst area / section (pixels x10^3^)			468±94	419±99		
cyst area / kidney area (%)	-	-	16.6±5.5	20.9±8.3		
Liver						
weight (g)	1.10±0.12	0.77±0.03	0.98±0.15	0.77±0.06		S
weight / body weight (g/100g)	4.06±0.13	3.98±0.06	4.25±0.09	3.95±0.13		
water content (%)	66.9±0.5	66.7±0.7	66.7±0.6	67.2±2.0		
cyst area / liver area (%)	-	-	0.24±0.20	0.07±0.03		
Feed intake (g/24h)	3.3±0.3	3.6±0.5	3.1±0.4	3.1±0.3		
Water intake (mL/24h)	3.5±0.5	4.1±0.4	3.2±0.3	3.9±0.8		
**n**	7	12	7	5		

Data from mice provided soy oil diets only. Values are mean±SE. D, disease; S, sex.

With respect to dietary oil effects, fish oil effects on renal disease were only observed in female mice: females given fish oil compared to either soy or flax oil had higher kidney weights and kidney water content. However, there were neither significant dietary oil effects on renal cyst area, nor on any liver parameters. All parameters in flax oil fed mice were similar to soy oil fed mice, with the exception of higher kidney weights in flax oil fed male mice. There were no dietary effects on feed intake, water intake or body weight (Table 2 and S1 and S2 Figs).

**Table 2:** Dietary oil and sex effects in *Pkd2*^WS25/-^ (diseased) mice.

	Soy Oil		Flax Oil		Fish Oil			
	Male	Female	Male	Female	Male	Female	P<0.05	
Body weight (g)	27.1±1.73	19.3±1.03	27.9±0.93	20.8±0.60	27.6±1.30	20.6±1.42		S
Kidney								
weight (g)	0.37±0.04^b^	0.27±0.03^b^	0.53±0.09^a^	0.34±0.03^b^	0.41±0.06^ab^	0.57±0.10^a^	I	
weight / body weight (g/100g)	1.37±0.11^b^	1.40±0.12^b^	1.89±0.33^b^	1.63±0.15^b^	1.44±0.14^b^	2.75±0.37^a^	I	
water content (%)	75.4±1.8^b^	74.8±1.1^b^	78.4±2.2^ab^	75.9±2.3^b^	75.5±0.6^b^	83.0±1.1^a^	I	
cyst area / section (pixels x10^3^)	468±94	419±99	774±292	566±131	599±145	998±230		
cyst area / kidney area (%)	16.6±5.5	20.9±8.3	23.0±5.9	24.4±5.5	22.6±5.1	32.3±7.2		
Liver								
weight (g)	0.98±0.15	0.77±0.06	1.18±0.06	0.89±0.02	1.18±0.06	0.93±0.06		S
weight / body weight (g/100g)	4.25±0.09	3.95±0.13	4.22±0.10	4.30±0.14	4.29±0.09	4.50±0.15		
water content (%)	66.7±0.6	67.2±2.0	65.9±1.4	66.8±1.1	65.6±0.4	66.1±1.0		
cyst area / liver area (%)	0.24±0.20	0.07±0.03	0.07±0.03	0.21±0.12	0.09±0.03	0.69±0.33		
Feed intake (g/24h)	3.1±0.4	3.1±0.3	2.6±0.1	3.2±0.4	2.3±0.2	4.2±0.6		
Water intake (mL/24h)	3.2±0.3	3.9±0.8	5.2±0.5	4.8±0.4	3.7±0.5	3.4±0.3		
**n**	7	5	5	7	8	4		

Values are mean±SE. With significant interaction effects, differing lower case superscript letters indicate significant simple effect differences between values. I, interaction; S, sex.

### PCK rats

PCK rats developed both kidney (Fig 1E–1H) and liver cysts (Fig 2E–2H) by 16 weeks of age. Renal cysts developed to a greater extent than hepatic cysts, with the cyst and lumen areas being ~ 4 times higher in kidneys compared to livers (Table 3). In contrast to *Pkd2*^WS25/-^ mice, fibrosis was detected in the livers but very little fibrosis was observed in the kidneys of PCK rats. The presence of cysts was reflected in higher kidney and liver weights and higher lumen and cyst area, as shown in normal and PCK rats given the control soy oil diet (Table 3). PCK rats also had higher urine protein levels and water intake, and lower feed intake and body weights than the normal rats, while serum creatinine, creatinine clearance, urine volume and urine pH were not different (Table 3).

**Table 3:** Disease effects in male PCK rats.

	Normal	Diseased
Body weight (g)	778±15	594±07*
Kidney		
weight (g)	3.8±0.1	4.4±0.1*
weight / body weight (g/100g)	0.49±0.01	0.75±0.01*
water content (%)	76.0±0.3	79.1±0.1*
lumen or cyst area area / section (pixels x 10^3^)	16±5	40±5*
lumen or cyst area / kidney area (%)	6.6±2.3	17.6±2.4*
Serum creatinine (mg/dL)	0.46±0.03	0.45±0.03
Creatinine clearance (mL/min)	26.8±4.4	20.8±2.7
Liver		
weight (g)	26.7±0.9	28.3±0.9
weight / body weight (g/100g)	3.44±0.11	4.78±0.16*
water content (%)	61.8±1.0	69.2±0.7*
fibrosis area / liver area (%)	0.2±0.0	3.5±0.3*
lumen or cyst area / liver area (%)	1.4±0.4	4.7±0.2*
Feed intake (g/24h)	30.0±1.9	24.1±0.6*
Water intake (mL/24h)	17.2±0.9	21.0±0.6*
Urine	10.3±0.9	10.1±0.9
pH	5.9±0.0	6.2±0.3
protein/creatinine (mg/mg)	0.9±0.2	14.7±3.8*
volume (mL/24h)	9.0±0.7	9.2±0.7
Mean arterial pressure (mmHg)	117.5±6.9	116.9±5.2
n	8	8

Data from mice provided soy oil diets only. Values are mean±SE.

* Significantly different from normal, P<0.05.

PCK rats given fish and flax oil compared to soy oil had larger kidneys with higher water content, kidney cyst and lumen area and creatinine clearance. Providing fish oil compared to both flax and soy oil resulted in larger livers. Rats given flax oil had higher urine pH than those given soy, but not fish oil. There were no dietary oil effects on body weight, feed intake, water intake, serum creatinine, urine protein, urine volume, blood pressure, liver cyst area or liver fibrosis (Table 4 and S3 and S4 Figs).

**Table 4:** Dietary oil and protein effects in diseased PCK rats.

	Soy Oil		Flax Oil		Fish Oil			
	Casein	Soy protein	Casein	Soy protein	Casein	Soy protein	P<0.05	
Body weight (g)	594±7	592±11	604±7	569±9	576±7	579±13		
Kidney								
weight (g)	4.4±0.1^B^	4.9±0.2	5.7±0.4^A^	5.2±0.2	5.4±0.3^A^	5.9±0.3	O	
weight / body weight (g/100g)	0.75±0.01^C^	0.83±0.03	0.87±0.02^B^	0.92±0.02	0.94±0.04^A^	0.99±0.04	O	P
water content (%)	79.1±0.1^B^	79.8±0.4	81.5±0.8^A^	81.0±0.4	80.4±0.5^A^	81.0±0.4	O	
lumen or cyst area/section (pixels x10^3^)	40±5^B^	48±5	67±8^A^	55±10	60±9^A^	62±6	O	
lumen or cyst area/kidney area (%)	17.6±2.4	18.4±1.9	22.3±2.5	21.2±3.6	21.7±3.0	20.7±1.6		
Liver								
weight (g)	28.3±0.9^B^	27.0±1.1	32.1±1.4^B^	24.7±0.7	35.5±2.9^A^	27.8±1.4	O	P
weight / body weight (g/100g)	4.78±0.16^B^	4.56±0.14	5.32±0.26^B^	4.34±0.09	6.16±0.49^A^	4.81±0.26	O	P
water content (%)	69.2±0.7^ab^	71.4±0.6^a^	70.1±0.4^ab^	68.8±0.4^b^	70.5±0.8^ab^	69.7±0.5^ab^	I
fibrosis area / liver area (%)	3.5±0.3	4.0±0.5	4.6±0.7	3.3±0.3	4.7±0.9	3.7±0.4		
lumen or cyst area / liver area (%)	4.7±0.2	3.9±0.5	6.1±0.8	5.4±1.1	6.0±0.9	4.9±0.8		
Feed intake (g/24h)	24.1±0.6	23.3±1.4	25.3±0.5	24.0±1.0	25.6±1.0	25.3±1.1		
Water intake (mL/24h)	21.0±0.6	27.4±1.8	22.8±1.1	26.5±1.09	23.0±1.8	29.3±1.6		P
Serum creatinine (mg/dL)	0.45±0.03^A^	0.44±0.02	0.49±0.04^A^	0.41±0.01	0.40±0.03^B^	0.39±0.04	O^§^	
Creatinine clearance (mL/min)	20.8±2.7^B^	23.4±1.7	25.5±2.8^A^	31.5±4.1	25.1±4.0^A^	37.1±4.1	O	P
Urine								
pH	6.2±0.3^B^	7.7±0.4	7.5±0.4^A^	8.3±0.3	7.3±0.6^AB^	7.9±0.4	O	P
protein / creatinine (mg/mg)	14.7±3.8	10.1±1.5	10.9±1.6	9.0±1.3	14.2±1.8	9.6±2.1		P
volume (mL/24h)	10.1±0.9	14.9±1.8	12.8±0.8	14.7±0.6	10.0±1.8	14.8±1.4		P
Mean arterial pressure (mmHg)	116.9±5.2	121.4±4.2	129.0±5.0	120.9±4.1	114.6±3.1	117.6±2.8		
**n**	8	8	8	8	6	8		

Values are mean±SE. With significant dietary oil effects, differing upper case superscript letters in casein columns indicate significant overall (casein and soy protein) differences between groups given different dietary oils. With significant interaction effects, differing lower case superscript letters indicate significant simple effect differences between values. I, interaction; O, oil; P, protein.

^§^ P = 0.057.

In contrast to *Pkd2*^WS25/-^ mouse models of ADPKD, providing soy protein compared to the casein protein found in the standard AIN93G diet resulted in higher relative kidney weights and lower liver weights in PCK rats. Dietary soy protein also resulted in higher water intake, creatinine clearance, urine pH and urine volume, and in lower urinary protein. Soy protein did not alter body weight, renal water content, renal cyst and lumen area, liver water content, liver cyst area, liver fibrosis, feed intake, serum creatinine or blood pressure (Table 4).

## Discussion

Overall, dietary interventions with oils enriched in omega-3 fatty acids provided early in the development of PKD displayed no benefits and possible negative effects on disease in both orthologous models of PKD studied. This lack of benefit in male PCK rats and in *Pkd2*^WS25/-^ mice is similar to the findings in female PCK rats [28]. In non-orthologous models, fish oil has conflicting effects, with generally protective effects observed in the Han:SPRD-*Cy* rat [23,24], while in the *pcy* mouse beneficial, detrimental and no effects have been observed [21,26,27]. With respect to liver effects, fish oil was recently shown to have no effects on liver cysts in female PCK rats, but complications due to cyst obstruction of the bile duct and hepatic vein were evident when these rats were given the fish oil diet [32]. Dietary flax oil also displayed no beneficial effects on disease in either orthologous PKD model, which contrasts with the beneficial effects observed in both the Han:SPRD-*Cy* rat and in *pcy* mice [19,21,22]. Although there were some effects of the oils containing n-3 fatty acids that were detrimental, these were small and not consistent in both models; as well, there were some potentially small positive effects, thus providing insufficient evidence to conclude that these dietary oils were harmful. Overall, these findings do not support dietary advice to increase dietary oils containing n-3 fatty acids for early treatment of PKD, and are consistent with a short-term study in human PKD that failed to demonstrate a beneficial effect of fish oil supplementation [33].

With respect to dietary protein source, there were no differences observed in the *Pkd2*^WS25/-^ mouse. In the PCK rats, although kidney and liver histology were not affected, water intake, creatinine clearance and urine pH were higher, and proteinuria was lower in soy protein fed rats. Similarly, dietary soy protein exhibited no benefits in female PCK rats provided soy protein diets at similar ages [28]. Increased water intake in the PCK rat is associated with reduction in kidney disease progression [34], and increased urine pH via citrate administration is associated with protection from disease in the Han:SPRD-*Cy* rat [35], possibly indicating that there may be benefits of this dietary intervention that had not yet been manifested, but require further study.

While these studies provide no supporting evidence for dietary advice in the early stages of PKD to increase soy protein or oils enriched in omega-3 fatty acids, it is important to determine whether interventions in later stages of disease would benefit from these treatments. Significant benefits of these identical dietary interventions were observed in non-orthologous models, in which disease progression is not only more rapid, but these animals also were terminated at a later stage of disease [10,11,21–23]. Studies in the Han:SPRD-*Cy* rat when these dietary soy protein, flax or fish oil interventions were initiated after the disease had progressed to the equivalent of approximately stage 3 CKD, dietary interventions also were not as effective, indicating that the intervention may have been too late in the disease process [14]. This suggests that there may be a window of opportunity during progressive cyst expansion in which dietary interventions may be more effective.

The amount of soy protein or omega-3 enriched oil also may influence the dietary effect, as the amounts used in the current diets are higher than what would be achievable in analogous human diets. However, the diets used herein were identical in this regard to the studies showing considerable slowing of disease progression in the non-orthologous Han:SRPD-*Cy* rat and *pcy* mouse models [11,21].

These results also may suggest that dietary interventions may be more effective in models of nephronophthisis (NPHP) compared to models of PKD. The *pcy* mouse is a model of NPHP3 [36], while the Anks6 protein mutated in the Han:SPRD-*Cy* rat appears to form complexes with NPHP proteins as well [37]. Nevertheless, these studies emphasize the need for studies in true models of PKD, and for replicating the results in multiple models, as no one model completely mimics the human form of the disease [38,39]. Investigation of possible sex effects also warrants further investigation.

## Conclusions

These studies show that dietary interventions with soy protein or omega-3 fatty acid enriched oils are not effective when administered during early PKD development in human orthologous models. However, evidence from non-orthologous models indicate that these dietary interventions may possibly be more effective during the progressive growth phase of cyst development.

## Supporting Information

S1 Fig Dietary oil and sex effects on kidney size in *Pkd2*^WS25/-^ (diseased) mice. There was a diet x sex interaction and differing lower case superscript letters indicate significant simple effect differences between values. Data from Table 2.
(PDF)

S2 Fig Dietary oil and sex effects on renal cyst area in *Pkd2*^WS25/-^ (diseased) mice. There were no significant diet or sex effects. Data from Table 2.
(PDF)

S3 Fig Dietary oil and protein effects on kidney size in diseased PCK rats. Significant diet effects are shown on figure. Data from Table 4.
(PDF)

S4 Fig Dietary oil and protein effects on renal cyst area in diseased PCK rats. There were no significant diet or sex effects. Data from Table 4.
(PDF)

S1 Table Details of experimental diets based on the AIN-93G diet for laboratory rodents [17]. (PDF)
