# Supplementary material for: Lack of Benefit of Early Intervention with Dietary Flax and Fish Oil and Soy Protein in Orthologous Rodent Models of Human Hereditary Polycystic Kidney Disease
Source: PLoS One. 2016 May 23;11(5):e0155790. doi: 10.1371/journal.pone.0155790 (PMC4877009; doi:10.1371/journal.pone.0155790)
Supplement: S1 Fig — There was a diet x sex interaction and differing lower case superscript letters indicate significant simple effect differences between values. Data from Table 2. (PDF) [file pone.0155790.s001.pdf]

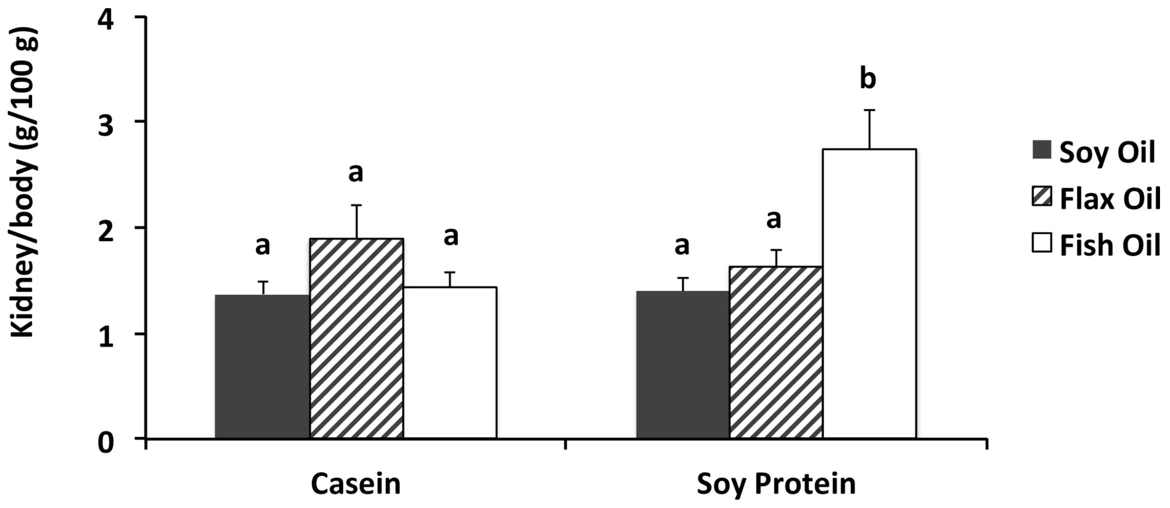

S1 Fig. Dietary oil and sex effects on kidney size in *Pkd2*$^{WS25/-}$ (diseased) mice. There was a diet x sex interaction and differing lower case superscript letters indicate significant simple effect differences between values. Data from Table 2.
