# Supplementary material for: Lack of Benefit of Early Intervention with Dietary Flax and Fish Oil and Soy Protein in Orthologous Rodent Models of Human Hereditary Polycystic Kidney Disease
Source: PLoS One. 2016 May 23;11(5):e0155790. doi: 10.1371/journal.pone.0155790 (PMC4877009; doi:10.1371/journal.pone.0155790)
Supplement: S2 Fig — There were no significant diet or sex effects. Data from Table 2. (PDF) [file pone.0155790.s002.pdf]

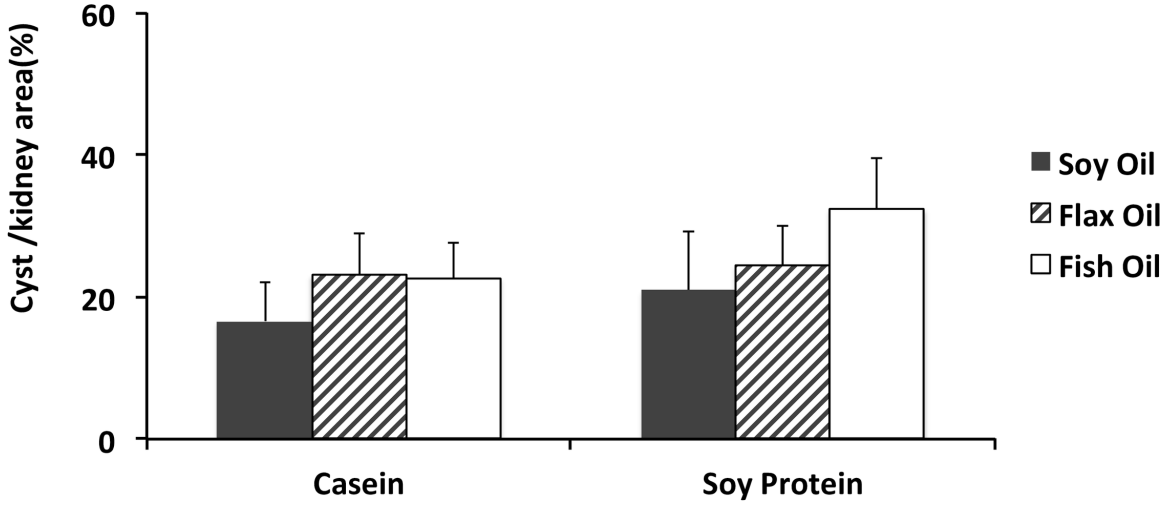

S2 Fig. Dietary oil and sex effects on renal cyst area in *Pkd2*$^{WS25/-}$ (diseased) mice.

There were no significant diet or sex effects. Data from Table 2.
