# Supplementary material for: Lack of Benefit of Early Intervention with Dietary Flax and Fish Oil and Soy Protein in Orthologous Rodent Models of Human Hereditary Polycystic Kidney Disease
Source: PLoS One. 2016 May 23;11(5):e0155790. doi: 10.1371/journal.pone.0155790 (PMC4877009; doi:10.1371/journal.pone.0155790)
Supplement: S3 Fig — Significant diet effects are shown on figure. Data from Table 4. (PDF) [file pone.0155790.s003.pdf]

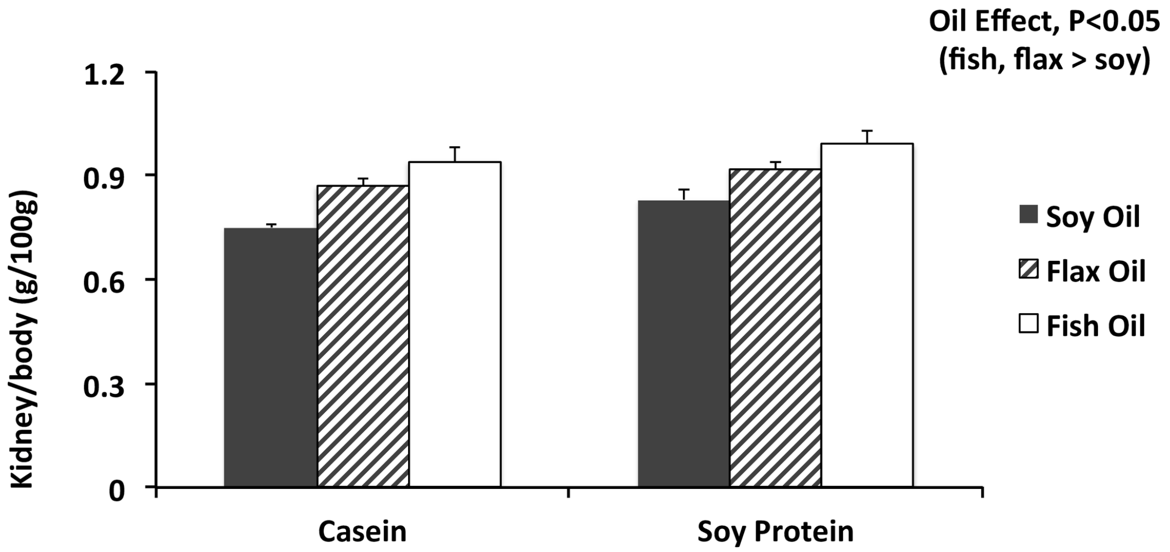

S3 Fig. Dietary oil and protein effects on kidney size in diseased PCK rats.

Significant diet effects are shown on figure. Data from Table 4.
