# Supplementary material for: Lack of Benefit of Early Intervention with Dietary Flax and Fish Oil and Soy Protein in Orthologous Rodent Models of Human Hereditary Polycystic Kidney Disease
Source: PLoS One. 2016 May 23;11(5):e0155790. doi: 10.1371/journal.pone.0155790 (PMC4877009; doi:10.1371/journal.pone.0155790)
Supplement: S4 Fig — There were no significant diet or sex effects. Data from Table 4. (PDF) [file pone.0155790.s004.pdf]

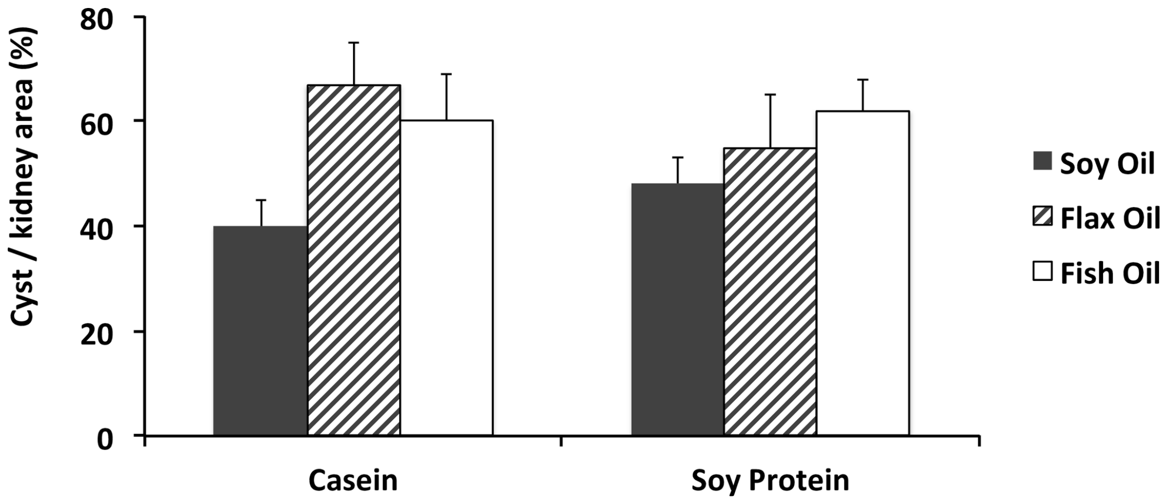

S4 Fig. Dietary oil and protein effects on renal cyst area in diseased PCK rats.

There were no significant diet or sex effects. Data from Table 4.
