# Supplementary material for: Lack of Benefit of Early Intervention with Dietary Flax and Fish Oil and Soy Protein in Orthologous Rodent Models of Human Hereditary Polycystic Kidney Disease
Source: PLoS One. 2016 May 23;11(5):e0155790. doi: 10.1371/journal.pone.0155790 (PMC4877009; doi:10.1371/journal.pone.0155790)
Supplement: S1 Table — (PDF) [file pone.0155790.s005.pdf]

S1 Table

Details of experimental diets based on the AIN-93G diet for laboratory rodents [17].

Protein source	Casein			Soy Protein		
Oil source	Soy oil	Flax oil	Fish oil	Soy oil	Flax oil	Fish oil
Ingredient			*g/kg diet*			
Casein (87% protein)	200	200	200	-	-	-
Soy protein (92% protein)	-	-	-	*189*	*189*	*189*
Soybean oil	70	*14*	*14*	70	*14*	*14*
Fish oil	-		*56*	-	-	*56*
Flax oil	-	*56*	-	-	*56*	-
Cornstarch	397.5	397.5	397.5	*408.5*	*408.5*	*408.5*
Dextrinized cornstarch	132	132	132	132	132	132
Sucrose	100	100	100	100	100	100
Fibre (cellulose)	50	50	50	50	50	50
Mineral mix (AIN93G)[1]	35	35	35	35	35	35
Vitamin mix (AIN93G)[1]	10	10	10	10	10	10
L-cystine	3	3	3	3	3	3
Choline bitartrate	2.5	2.5	2.5	2.5	2.5	2.5
Tert-butylhydroquinone[2]	0.014	0.014	0.014	0.014	0.014	0.014

[1]Details of the mineral and vitamin mix are found in [17].

[2]Antioxidant added to the oils.

The shaded numbers are those that are modified from the control diet (casein protein, soy oil) to create the experimental diets. Diet ingredients were purchased from Dyets Inc. (Bethlehem, PA) and Harlan Teklad (Madison, WI).
